# Modelling falls in Parkinson’s disease and normal ageing in mice using a complex motor task

**DOI:** 10.1177/23982128221088794

**Published:** 2022-03-22

**Authors:** Megan G. Jackson, Laura J. Brennan, Emily J. Henderson, Emma S. J. Robinson

**Affiliations:** 1 School of Physiology, Pharmacology & Neuroscience, University of Bristol, Bristol, UK; 2 Population Health Sciences, Bristol Medical School, University of Bristol, Bristol, UK; 3 Royal United Hospitals Bath, NHS Foundation Trust, Bath, UK

**Keywords:** Ageing, Parkinson’s disease, falls, cognition, mouse model, gait

## Abstract

Falls resulting from multifactorial deficits are common in both normal ageing and Parkinson’s disease. Resultant injuries can lead to increased hospitalisation and excess mortality. As the disease progresses, gait and balance deficits are relatively refractory to dopaminergic treatments suggesting another system is involved. Attentional impairment is a significant risk factor for falls, and disruption to both the cortical cholinergic system and striatal dopaminergic system increases falls in rats undergoing a complex motor task with high attentional load. However, it is unclear whether this translates to mice and whether normal ageing induces similar deficits. In this study, we use a complex motor task to test the effects of acute dopaminergic and cholinergic antagonism using alpha-flupentixol and scopolamine, respectively, in mice. We also test the effects of normal ageing on complex motor performance and whether these changes are sensitive to a clinical dose of the non-steroidal anti-inflammatory Rimadyl. Consistent with previous work, we show that cholinergic but not dopaminergic antagonism impaired task performance. However, a combined approach did not potentiate the deficit beyond observed with cholinergic antagonism alone. We also show that task performance is impaired in aged mice relative to younger controls, and that Rimadyl reduces number of foot slips in an age-specific manner. Overall, these data support prior work showing the importance of the cholinergic system in falls. The studies in aged mice found age-related impairments and a role for inflammation but did not find evidence of an interaction with attentional load, although only one manipulation was tested.

## Introduction

Falls in older people and those with neurodegenerative conditions represent a major public health challenge. The aetiology of falls is multifactorial, but gait and balance dysfunction leading to postural instability are significant risk factors (Salzman, 2010). Falls are associated with increased hospitalisation and mortality, institutionalisation and poorer quality of life for both the patient and their caregiver (Dunne et al., 2014; Kenny et al., 2017; Stel et al., 2004). The prevalence of falls is particularly high in older adults, with 30% of >65s and 50% of >80s suffering a fall annually (NICE, 2013). These figures are greater still in Parkinson’s disease (PD) patients. A study has shown that 50% of PD patients have had at least one fall, and are nine-fold more at risk of suffering recurrent falls (Bloem et al., 2001). Falls are therefore of clear clinical importance, but treatment approaches are limited, and with disease progression are increasingly refractory to the mainstay of PD therapeutics, levodopa. This lack of clinical effect suggests that motor deficits leading to falls are caused by a mechanism beyond dopaminergic depletion (Lim et al., 2009).

Falls and attentional deficits are closely linked, as normal gait utilises attention rather than being an automated motor activity. When attention is divided, for example, by dual tasking, gait becomes slowed in healthy adults and children (Yogev-Seligmann et al., 2008). In tasks requiring both sustained and divided attention, impairments in attention robustly predict falls and changes in gait in both the healthy aged and patients with PD (Allcock et al., 2009; Lord et al., 2010; O’Halloran et al., 2011). These attentional deficits are thought to be driven by cholinergic depletion in the basal forebrain (Sarter et al., 2014). As such, a dual-system driving complex motor control has been posed, whereby cortical cholinergic inputs drive the attentional processing of movement-related cues, and feeds this information to the striatal circuitry to select and sequence complex movement (Sarter et al., 2014). In contrast to dopamine-specific models of PD, deficits in both the cholinergic and the dopaminergic system may be required to dismantle this cognitive-motor interaction, and more accurately model falls seen in PD and normal ageing. Indeed, depletion of both the cholinergic and dopaminergic systems has been reported in both PD and, to a lesser degree, normal ageing (Bäckman et al., 2006; Bartus et al., 1982; Bohnen and Albin, 2011). This approach may extend to other neurodegenerative diseases where the cholinergic system is affected, such as Alzheimer’s disease, where falls are also commonly reported (Horikawa et al., 2005).

There are several different tasks that have been used to measure rodent gait, posture and balance, including the raised beam test and rotarod (Schönfeld et al., 2017). However, to capture the impact of attentional impairment on falls, the task must carry a sufficient attentional load. Work by Kucinski et al. (2013) designed a complex motor task to assess the interaction between attention, gait and their impact on falls. The Michigan Complex Motor Control Test (MCMCT) requires the rodent to navigate a 2-m long complex surface which rotates, which is thought to translate to how a person with PD must navigate complex environments and uneven walking surfaces as part of daily life. Using rats, it was found that cholinergic but not dopaminergic depletion increased falls in this task, and when cholinergic and dopaminergic depletion were combined, the complex motor control deficit was potentiated. These findings provide key evidence that falls in PD are likely to result from a dual-system deficit, and should be considered in the development of therapeutics. However, it is not clear whether these findings in rat models translate to mice, where genetic models are still more readily developed. The effect of normal ageing, where falls are also prevalent, has not previously been described.

As the pathophysiology of PD involves degeneration of both the cholinergic and dopaminergic systems, and there is evidence to suggest it is an interaction of both of these systems that modulates complex motor control, the present experiment was designed to test the effects of acute cholinergic and dopaminergic antagonism on complex motor control using a balance beam motor control task in mice. This was done using systemic administration of the muscarinic receptor antagonist scopolamine (Renner et al., 2005), and the dopaminergic receptor antagonist alpha-flupentixol (Mahapatra et al., 2014). It is important to understand changes related to normal ageing to help understand changes in age-related neurodegenerative disease, such as PD. Therefore, we tested the effects of normal ageing on task performance. In addition, we used an ethologically relevant olfactory distractor to assess the interaction between motor control and increased attentional load in normal ageing. To reveal whether any potential age-related impairments were driven by inflammation of the joints, we also tested whether motor performance was affected by inflammatory processes by pre-treating animals with an non-steroidal anti-inflammatory drug (NSAID).

## Methods

### General husbandry

A total of 46 mice were used across the three studies described below. Mice were kept under temperature-controlled conditions, 20–21°C and a 12:12 h reverse light–dark cycle (lights on 20:15). Water and food (standard lab chow, Purina, UK) were provided ad libitum. Cages were enriched with a cardboard tube, wooden chew block and small house. All behavioural testing was carried out during the animal’s active phase under red lighting. All experiments were conducted in accordance with the Animals (Scientific Procedures) Act UK, 1986 and the University of Bristol guidelines.

### Equipment

**Figure 1.**
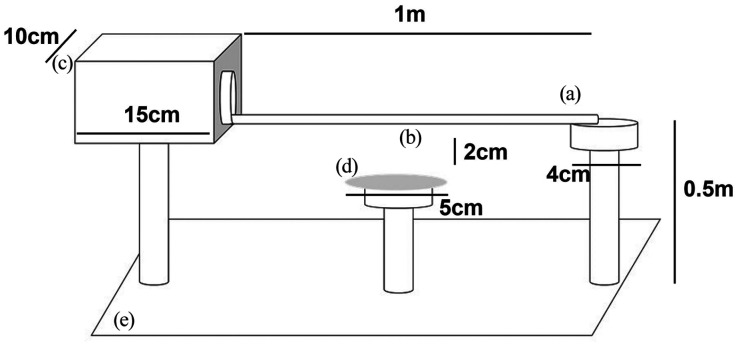
Motor task apparatus. (a) Starting podium, (b) traversal rod, (c) goal box, (d) olfactory distractor platform and (e) soft landing substrate.

The motor task equipment comprised three elements (Figure 1). A 6-cm, circular start podium and an 8-cm goal box made of clear Perspex at the opposite end. This was connected by a 1-m long plastic rod. Three different rod diameters (10, 8 and 6 mm) were used for testing. Different sized rods were used to modulate attentional load, with the widest rod hypothesised to represent the easiest to traverse. Two clamp stands supported the goal box and start podium. The rod was suspended 0.5 m above the table. Plastic bubble wrap was placed underneath the rod for protection when mice fell from the rod. Reward pellets (Test Diet, USA, AIN-76A Rodent Tablet 20 mg) were placed in the goal box to reward complete traversal.

### Training

Initial habituation consisted of placing mice at incremental distances along the rod and allowing them to traverse to the goal box and collect reward pellets. All cohorts were trained starting on the 10-mm rod. The five stages of training are outlined in Table 1.

**Table 1.** Stages of training for both cohorts of mice.

Stage	Description	Criteria for progression
1	Placed halfway along the 10-mm rod	3 successful half traversals of the rod
2	Placed halfway along the 10-mm rod and then placed on starting podium	5 successful half traversals and 1 successful full traversal of the rod
3	Placed on starting podium	5 successful full traversals of the widest rod
4	Placed on starting podium	5 successful full traversals of next rod size down; 8 mm
5	Placed on starting podium	5 successful full traversals of next rod size down; 6 mm

## The complex motor task design

Number of falls, foot slips and traversal time were the primary outcome measures and have been previously used to quantify complex motor behaviour (Kucinski et al., 2013). Foot slips and traversal time have previously been shown to correlate with number of falls (Kucinski et al., 2015). A foot slip was defined as one foot losing contact with the rod. Each foot slip was equal to the number of paws leaving the rod (hind and front limbs). If both hindlimbs lost contact with the rod but the mouse recovered and returned to the rod, this was counted as two foot slips. A fall was defined as all four feet leaving the rod. Foot slips and falls were only measured once there was full initiated movement from the start podium, with all four paws on the rod. Traversal time (in seconds) was recorded; the timer was started when all four feet left the start podium and stopped when all four feet were within the goal box. If more than 25 s elapsed and a traversal was not completed, the animal was removed, and this was an omitted trial. One trial was equal to one full traversal; if the mouse fell, they were re-placed on the start podium and a new trial was started. Traversal time, number of foot slips and number of falls were recorded manually and then averaged over five traversals for the anti-cholinergic/dopaminergic manipulation studies, and three trials for the ageing studies to reduce potential non-specific effects of fatigue. A within-subject fully counterbalanced design was used in all studies. During testing, the experimenter was blind to treatment but not to age group due to obvious phenotypic differences. Traversal time, foot slips and falls were combined into a composite score (Table 2) by adding up their scores. This approach allowed for individual measures of complex motor control to be analysed in case of dissociable effects, and to also integrate these measures into an overall readout of complex motor control.

**Table 2.** Composite score.

Falls		Foot slips		Traversal time	
Number of falls	Score	Number of foot slips	Score	Mean score + seconds	Score
0	0	0	0	<Mean to mean + 0.5 s	0
1	5	1–2	1	Mean + 0.5–1 s	1
2	10	3–4	2	Mean + 1–1.5 s	2
⩾3	Excluded	5–6	3	Mean + 1.5–2 s	3
		>6	4	Mean + 2–2.5 s	4
				Mean + >2.5 s	5

### Study 1 – the effects of ageing and olfactory distraction on complex motor performance

#### Subjects

N = 12 C57Bl/6J male mice were obtained from Charles River aged 8 weeks. At the time of testing, they were approximately 6 months old with a free-feeding weight of 24–31 g. N = 12 C57Bl/6J male mice were kindly provided by Eli Lilly at the age of 15 months and were 18 months old with a free-feeding weight of 34–39 g at time of testing. C57bl/6J mice were used in studies 1 and 2 because this strain is commonly used in ageing work, due to their longevity. Males were used in this case as it has been suggested that male mice are less likely to become frail, and thus less likely to increase the likelihood of non-specific impairment in the task, compared to female mice (Baumann et al., 2019). Mice were group housed in trios on arrival; however, we noticed considerable in-fighting in the aged group, so upon advice from our named veterinary surgeon (NVS), the mice were singly housed for their welfare. The younger mice were also singly housed to keep housing conditions consistent within the study. Therefore, mice were singly housed for the duration of the study. Aged mice were checked daily for signs of ill health or overt motoric changes that would influence task performance. In this case, mice were removed from the experiment; however, this did not occur.

Mice were trained as above and completed three traversals of an 8-mm rod. Performance was then observed under the influence of an olfactory distractor. Filter paper was placed in cages of female mice overnight to collect scent, and then placed 2 cm below the rod to be used as an olfactory distractor. Olfactory distractors were not in the vertical line of sight of the animals. Ethanol was used to clean the rod between subjects. Falls, foot slips and traversal time were recorded. Mice that fell 3 times from the rod were excluded (n = 2 aged mice).

### Study 2 – the effects of a non-steroidal anti-inflammatory drug on complex motor performance

#### Subjects

To assess whether results could be reproduced at a more advanced age, and whether deficits were modulated by inflammatory processes, mice in the above ageing study were used again, with aged mice at 23 months old and younger mice at 11 months old at time of experimentation. One aged mouse died before this experiment.

Due to a 5-month gap between studies, mice were given a refresher training session where they completed three half traversals of a 10-mm rod and three complete traversals from start platform to goal box to receive a food reward. All mice successfully completed this stage of training. For the test, mice were dosed sub-cut with either a clinical dose of Rimadyl advised by the NVS (2 mg/kg) (50 mg/ml, vet formulation) dissolved in saline or saline vehicle 1 h before the task. Mice traversed the full length of the 10-mm rod 3 times. Mice that fell 3 times from the rod were excluded; however, this did not occur. The study was within-subject and counterbalanced by age and drug dose.

### Study 3 – anti-dopaminergic and anti-cholinergic dose–response studies

#### Subjects

N = 22 CD1 female mice were used for the following dose–response studies. N = 11 mice were used for the alpha-flupentixol study and a new cohort of N = 11 were used for the scopolamine and combination study. Mice were obtained from Charles River Laboratories aged 12 weeks. They were housed in groups of four and had a free-feeding weight of 23–30 g. Female mice were selected for the pharmacological studies as it has previously been shown that female rats show a relatively greater degree of complex motor control compared to males, and were more sensitive to the therapeutic effects of an M1 allosteric modulator following cholinergic lesioning. Thus, using females may provide more information about the effects of pharmacological manipulation on complex motor control (Kucinski and Sarter, 2021). In addition, given our previous problems with males fighting and ageing was not the primary objective, using females was also seen as a refinement as the animals could be group housed.

Following training, the effects of various rod sizes and distractor effects on falls, foot slips and traversal time were tested in a series of acute pharmacological dose–response manipulations. Studies used scopolamine and alpha-flupentixol, and a combination of the two (Table 3). Scopolamine (Tocris Bioscience, UK) and alpha-flupentixol (Sigma-RBI, USA) were administered via intraperitoneal (IP) injection 1 h prior to the experiment. Drugs were dissolved in 0.9% sterile saline and administered at a dose volume of 10.0 ml/kg. Mice traversed the 8-mm rod 5 times. Each drug study was carried out as an independent, within-subject experiment with at least a 48-h washout period between studies.

**Table 3.** Dose–response study details.

Drug	Dose (mg/kg)
Scopolamine	0, 1.0, 3.0
Alpha-flupentixol	0, 0.1, 0.3
Scopolamine + Alpha-flupentixol	1.0 + 0.3

### Statistical analysis

Sample sizes were based on an estimated effect size from previous work testing the effects of pharmacological intervention in a similar attentional-motor task. We used similar sample sizes as this previous study because we expected a similar effect size (Kucinski and Sarter, 2021). For the ageing studies, performance on the rod was averaged over three trials. A repeated-measures (RM) two-way analysis of variance (ANOVA), with age as a between-subjects factor and distractor/drug dose as a within-subject factor, was used. An exclusion criterion was set where mice that fell from the rod 3 times were excluded, as meaningful information about their traversal time and foot slips could not be collected. For the dose–response studies, performance on the rod was averaged over five trials. An RM two-way ANOVA with dose and rod size as within-subject factors was used. Where a main effect was observed (p < 0.05), appropriate pairwise comparisons were used. Where a trend was observed (p < 0.1), this was stated but not further analysed. For the anti-cholinergic and anti-dopaminergic manipulations, performance on the rod was averaged over five trials. In studies 2 and 3, falls were not statistically analysed due to limited number of falls, and instead average number of falls was reported and is incorporated in the composite score. Where sphericity was not assumed, the Huynh–Feldt corrected value was used. Exact p values are reported except for the cases where p < 0.0001, where this was reported instead. All statistical analysis was completed using IBM SPSS Statistics 24. Graphs were formatted using GraphPad Prism v.7.

## Results

### Aged mice show deficits in rod traversal performance that are removed in the presence of an olfactory distractor

**Figure 2.**
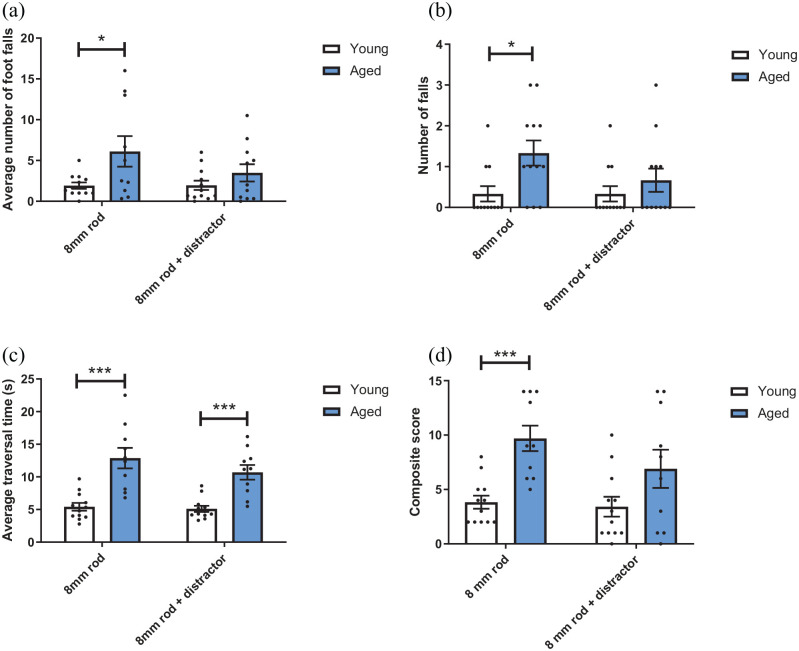
Aged mice show deficits in complex motor performance that are removed in the presence of a distractor. Aged and young mice traversed an 8-mm, 1-m long rod in the presence and absence of an olfactory distractor. (a) Aged mice made a greater number of foot slips compared to younger mice in the absence of a distractor (p < 0.05, RM two-way ANOVA with pairwise comparisons). (b) Aged mice fell more than younger mice when traversing a rod in the absence of a distractor (p < 0.05). (c) Aged mice take longer to traverse the rod than younger mice in both the presence and absence of a distractor (p < 0.001). (d) Aged mice had a greater composite score than younger mice in the absence of a distractor (p < 0.001, pairwise comparisons). Bars are mean ± SEM with data points overlaid. N = 12 young mice and n = 10–12 aged mice. *p < 0.05, ***p < 0.001.

There was a main effect of age group on average foot slips during traversal of the rod (F(1,20) = 5.084, p = 0.036). Post hoc analysis revealed that in the absence of an olfactory distractor, aged mice made more foot slips than younger mice (p = 0.027) but this difference disappeared in the presence of the distractor (p > 0.05). There was no main effect of distractor, or a distractor presence*group interaction (p > 0.05) (Figure 2(a)).

There was a main effect of age on falls during rod traversal, where aged mice fell more times than younger mice (F(1,22) = 7.184, p = 0.014). Post hoc analysis revealed that aged mice fell more than younger mice in the absence of a distractor (p = 0.013) but not in the presence of a distractor (p = 0.339). There was no main effect of distractor or distractor*group interaction (p > 0.05) (Figure 2(b)).

Analysis of traversal time showed a main effect of age on traversal time (F(1,19) = 31.154, p < 0.0001). Aged mice took longer to traverse the rod in both the presence and absence of a distractor (p = 0.0006 and 0.0009, respectively). However, there was no distractor presence*group interaction (p > 0.05) or main effect of olfactory distractor, though there was a trend (F_(1,19)_ = 3.371, p = 0.082) (Figure 2(c)).

Analysis of overall composite score showed no main effect of distractor presence or distractor presence*group interaction (p > 0.05). However, there was a main effect of age (p = 0.00025). Post hoc pairwise comparison revealed that aged mice had a higher composite score than younger mice in the absence of a distractor (p = 0.00015) but not in the presence of a distractor (p = 0.079) (Figure 2(d)).

### Rimadyl reduces number of foot slips but not traversal time or falls in aged mice

**Figure 3.**
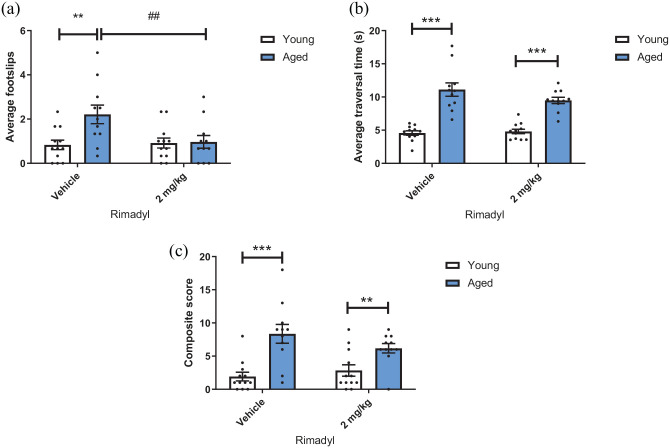
Rimadyl reduces foot slips in aged mice but not falls or traversal time. Mice traversed a 10-mm, 1-m long rod and were dosed with either 2-mg/kg Rimadyl or saline vehicle. (a) Aged mice made a greater number of foot slips than younger mice under vehicle conditions. Rimadyl reduced the number of foot slips in aged mice but not young mice (p < 0.01, RM two-way model ANOVA with post hoc pairwise comparisons). (b) Under both vehicle and Rimadyl conditions, aged mice took longer to traverse the rod than younger mice (p < 0.001). (c) Aged mice had a greater composite score than younger mice in both vehicle and Rimadyl conditions (p < 0.001 and <0.01, respectively, pairwise comparisons). Bars are mean ± SEM with data appoints overlaid. N = 12 younger mice and n = 11 aged mice. **p < 0.01, ***p < 0.001 (between-subject). #p < 0.05, ##p < 0.01 (within-subject).

There was a main effect of drug and a drug*age interaction on average foot slips (F_(1,21)_ = 5.542, p = 0.028 and F_(1,21)_ = 7.251, p = 0.014, respectively). There was also a main effect of age (F_(1,21)_ = 4.581, p = 0.044). Post hoc analysis revealed that Rimadyl decreased the number of foot slips in the aged group but not the younger group (p = 0.002). Under vehicle conditions, aged mice made more foot slips when traversing a rod compared to the younger group (p = 0.007). However, under Rimadyl conditions, this difference disappears (p > 0.05) (Figure 3(a)).

As previously described in the ‘Methods’ section, falls in this experiment were not statistically analysed due to limited number of falls, and instead average number of falls are reported in Table 4.

**Table 4.** Average falls under vehicle and Rimadyl treatment in young and aged groups.

Age group	Drug dose (Rimadyl)	
	Vehicle	2 mg/kg
Young	0.08	0.25
Aged	0.45	0.18

There was no main effect of drug on traversal time (p > 0.05), but there was a trend towards a drug*age interaction (F_(1,21)_ = 3.040, p = 0.096). There was also an effect of age on traversal time (F_(1,21)_ = 78.650, p < 0.0001). Post hoc analysis revealed that under both vehicle and Rimadyl conditions, aged mice were slower to traverse the rod than the younger group (p < 0.0001) (Figure 3(b)).

There was no main effect of drug on composite score (p > 0.05), but there was a trend towards a drug*age interaction (F_(1,21)_ = 3.073, p = 0.094). There was also a main effect of age (F_(1,21)_ = 23.845, p < 0.0001). Post hoc analysis revealed that under both vehicle and Rimadyl conditions, aged mice had a higher composite score than younger mice (p = 0.00036 and 0.007, respectively) (Figure 3(c)).

### Scopolamine impairs complex motor performance

**Figure 4.**
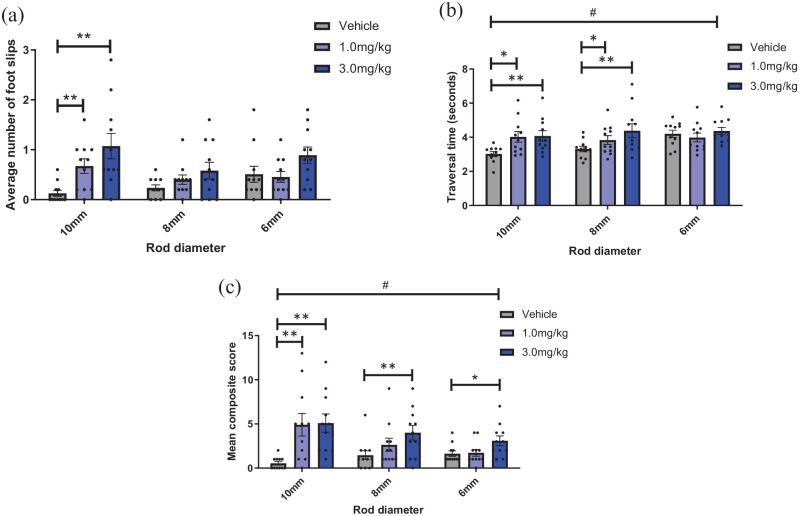
Scopolamine impairs motor performance on a complex motor task. Mice were dosed with either vehicle, 1.0- or 3.0-mg/kg scopolamine and completed the complex motor task. (a) 1.0- and 3.0-mg/kg scopolamine increased foot slips on the 10-mm rod (p < 0.01, RM two-way ANOVA with pairwise comparisons). (b) 1.0- and 3.0-mg/kg scopolamine increased traversal time on the 10-mm rod and 3.0 mg/kg increased traversal time on the 8-mm rod (p < 0.05). (c) 1.0- and 3.0-mg/kg scopolamine increased composite score on the 10-mm rod (p < 0.01) and 3.0-mg/kg scopolamine increased composite score on the 8-mm rod (p < 0.01) and the 6-mm rod (p < 0.05). Bars are mean ± SEM with data points overlaid. N = 11. *p < 0.05, **p < 0.01, #p < 0.05 (within-subject effect of rod size).

There was a main effect of scopolamine on number of foot slips (F_2,22_ = 7.575, p = 0.016) and a drug*rod size interaction (F_4,40_ = 5.186, p = 0.004) . Post hoc pairwise comparisons revealed that both 1.0- and 3.0-mg/kg scopolamine increased number of foot slips compared to vehicle on the 10-mm rod (p = 0.003 and 0.003, respectively). There was no main effect of rod size (p > 0.05) (Figure 4(a)).

Average falls under scopolamine treatment are reported in Table 5.

**Table 5.** Average falls under scopolamine treatment.

Rod diameter (mm)	Drug dose (scopolamine)		
	Vehicle	1.0 mg/kg	3.0 mg/kg
10	0	0.27	0.18
8	0.09	0.09	0.18
6	0	0	0

There was a main effect of scopolamine on traversal time (F_2,20_ = 4.823, p = 0.040), a drug*rod size interaction (F_4,40_ = 4.788, p = 0.003) and a main effect of rod size (F_2,20_ = 8.486, p = 0.011). Post hoc comparison showed that 1.0- and 3.0-mg/kg scopolamine increased traversal time compared to vehicle on both the 10-mm rod (p = 0.009 and 0.002, respectively) and the 8-mm rod (p = 0.024 and 0.004, respectively). There was no effect of scopolamine of traversal time on the 6-mm rod (p > 0.05) (Figure 4(b)).

Finally, there was a main effect of scopolamine on composite score (F_2,20_ = 5.848, p = 0.010). There was also a drug*rod size interaction (F_4,40_ = 5.646, p = 0.001) and a main effect of rod size (F_2,20_ = 4.483, p = 0.025). Post hoc pairwise comparison revealed 3.0 mg/kg increased mean composite score on the 10-, 8- and 6-mm rods (p = 0.002, 0.001 and 0.031, respectively), and 1.0 mg/kg resulted in a higher composite score on the 10-mm rod (p = 0.008) (Figure 4(c)).

### Alpha-flupentixol has no effect on complex motor performance

**Figure 5.**
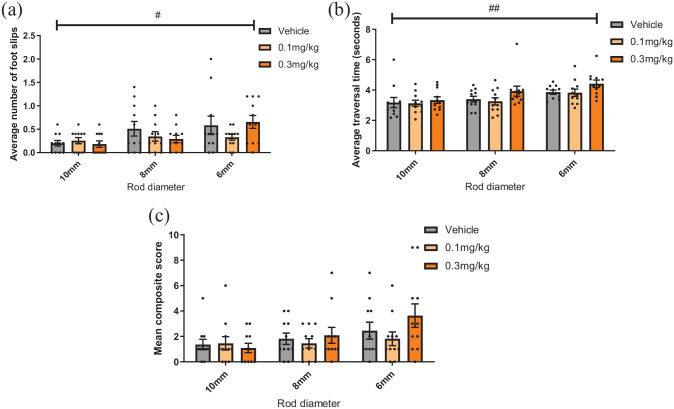
Alpha-flupentixol has no effect on motor performance in a complex motor task. Mice were dosed with either vehicle, 0.1- or 0.3-mg/kg alpha-flupentixol and completed a complex motor task. (a) Alpha-flupentixol had no effect on foot slips (p > 0.05, RM two-way ANOVA). (b) Alpha-flupentixol had no effect on traversal time (p > 0.05). (c) Alpha-flupentixol had no effect on composite score (p > 0.05). Bars are mean ± SEM with data points overlaid. N = 11. #p < 0.05, ##p < 0.01 (within-subject effect of rod size).

There was no main effect of alpha-flupentixol on number of foot slips (p > 0.05) and no drug*rod size interaction (p > 0.05). There was however an effect of rod size on the number of foot slips (rod, F_2,20_ = 5.628, p = 0.012) (Figure 5(a)).

Average falls under alpha-flupentixol treatment are reported in Table 6.

**Table 6.** Average falls under alpha-flupentixol treatment.

Rod diameter (mm)	Drug dose (alpha-flupentixol)		
	Vehicle	0.1 mg/kg	0.3 mg/kg
10	0	0.091	0
8	0	0	0.091
6	0.182	0.091	0.182

There was a main effect of alpha-flupentixol on traversal time (drug, F_2,20_ = 4.823, p = 0.040), a main effect of rod size (F_2,20_ = 63.285, p = 0.001) but no drug*rod size interaction (p > 0.05). However, post hoc analysis showed no effect of drug compared to vehicle on any of the three rod sizes (Figure 5(b)).

There was no main effect of treatment on composite score (F_2,20_ = 1.932, p = 0.171) or a drug*rod interaction (F_4,40_ = 1.595, p = 0.194) but there was a trend towards a main effect of rod size (F_2,20_ = 3.480, p = 0.050) (Figure 5(c)).

## A combination of alpha-flupentixol and scopolamine impairs complex motor performance

**Figure 6.**
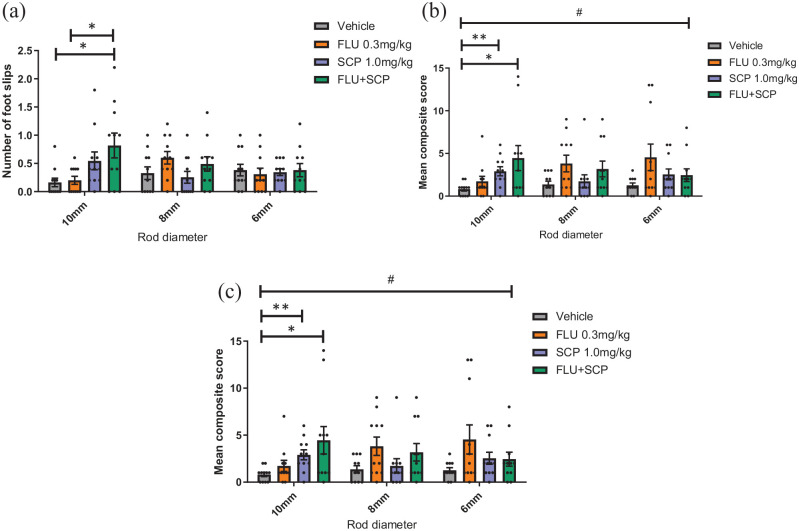
A combination of scopolamine and alpha-flupentixol impairs motor performance on a complex motor task. Mice were dosed with either vehicle, 0.3-mg/kg alpha-flupentixol, 1.0-mg/kg scopolamine or a combination of the two. (a) A combination of alpha-flupentixol and scopolamine increased number of foot slips compared to vehicle and alpha-flupentixol (p < 0.05, RM two-way ANOVA with post hoc pairwise comparisons). (b) Drug treatment did not affect traversal time (p > 0.05). (c) Scopolamine and scopolamine in combination with alpha-flupentixol increased the mean composite score on the 10-mm rod (p < 0.01 and <0.05, respectively). Bars are mean ± SEM with data points overlaid. N = 11. *p < 0.05, **p < 0.01, #p < 0.05 (within-subject effect of rod size).

There was no main effect of treatment on foot slips, though there was a trend (F_3,30_ = 2.932, p = 0.060). However, there was as a drug*rod interaction (F_6,60_ = 3.556, p = 0.004) but no main effect of rod size (p > 0.05). Post hoc analysis showed that treatment with scopolamine and alpha-flupentixol combined increased number of foot slips on the 10-mm rod compared to vehicle (p = 0.022). The combined treatment also increased number of foot slips compared to alpha-flupentixol alone (p = 0.028). These effects were not seen on the 8- and 6-mm rods (p > 0.05) (Figure 6(a)).

Average falls under scopolamine, alpha-flupentixol and combined treatment are reported in Table 7.

**Table 7.** Average falls under scopolamine, alpha-flupentixol and a combined approach.

Rod diameter (mm)	Drug dose			
	Vehicle	Scopolamine (1.0 mg/kg)	Alpha-flupentixol (0.3 mg/kg)	Scopolamine/alpha-flupentixol combined
10	0	0.091	0.091	0.182
8	0	0.091	0.091	0
6	0	0.091	0.282	0.091

Traversal time was not affected by any of the treatment conditions (p > 0.05) (Figure 6(b)).

There was a main effect of drug on composite score (F_3,30_ = 3.051, p = 0.044), a drug*rod size interaction (F_6,60_ = 2.626, p = 0.046) and a main effect of rod size (F_6,60_ = 2.626, p = 0.046). Post hoc pairwise comparisons revealed that scopolamine treatment and scopolamine plus alpha-flupentixol increased composite score compared to vehicle (p = 0.001 and 0.034, respectively) on the 10-mm rod (Figure 6(c)).

## Discussion

Using this balance beam task, we demonstrate that task performance, measured by foot slips, falls, traversal time and a composite score, is reduced in healthy aged mice. When aged and young mice are presented with an olfactory distractor, the age-related differences in foot slips and falls disappear suggesting an interaction with attentional processes although not in the predicted direction. A clinical dose of the NSAID Rimadyl specifically reduces foot slips in aged but not young mice. An acute dose of the anti-muscarinic drug scopolamine impairs complex motor performance, inducing an increase in foot slips, traversal time and composite score. Administration of the anti-dopaminergic drug alpha-flupentixol had no effect on complex motor performance. A combination of both an anti-muscarinic and anti-dopaminergic drug did not potentiate impairments relative to cholinergic manipulation alone. Here, we consider how these findings relate to previous work and the overall suitability of this design as a method for assessing complex motor control and their interaction with attentional processes in mouse models.

## Normal ageing reduces task performance

As a group, naturally aged mice showed an increased number of foot slips, falls and a longer traversal time compared to younger mice. This impairment in complex motor performance is consistent with clinical data, showing that older adults show slowing in movement, and difficulties in co-ordination, balance and gait (Seidler et al., 2002, 2010). There is comparatively little work in healthy aged mice; however, a reduction in gait velocity on a rotarod has recently been reported (Yanai and Endo, 2021), which may reflect a reduction in complex motor control. The present findings therefore provide some evidence that the complex motor task utilised in this study is sensitive to age-related impairments and could be used to investigate potential therapeutics in future studies. There is evidence for age-related disruption to cholinergic and dopaminergic modulation, including decreased muscarinic receptor density and functional reactivity of cholinoreceptive neurons. In turn, these deficits have been linked with cognitive and attentional deficits, providing a potential mechanism for age-related deficits in this task (Bäckman et al., 2006; Bartus et al., 1982; Düzel et al., 2010). Of note, there is some variation within the aged group in this study, perhaps suggesting differing levels of age-related dopaminergic/cholinergic degeneration, or different levels of joint inflammation which may in turn impact performance. However, we present only behavioural data so we cannot make any firm conclusions about a potential mechanism for this variation in the aged mice.

Rimadyl reduced the number of foot slips in the aged but not young mice, suggesting Rimadyl had an age-specific effect that may be related to inflammation. Of note, the therapeutic effect of Rimadyl was specific to foot slips. This suggests that foot slips and traversal time are dissociable measures of complex motor control. It should be noted that the complex motor task inherently has a rewarding and aversive component to it. The mice were not food restricted. However, the goal box contains palatable reward, and provides cover to an otherwise exposed environment. Previous work has shown that aged mice show a reduction in motivation, reward sensitivity and anxiety-like behaviour which may affect desire to reach the goal box (Jackson et al., 2021). Thus, traversal time but not foot slips may be sensitive to changes in goal-directed/habitual movement in this task.

An olfactory distractor was used as an ethologically relevant method to distract attentional resource away from the primary complex motor task and thus reduce supervision of gait, balance and complex movement, with the expectation this distractor would potentiate any age-related deficit in complex motor performance. However, in the presence of the olfactory distractor, the age-related increase in foot slips and falls disappeared. This contrasts with previous work showing that the addition of a doorframe distractor to the MCMCT increased fall number in rats (Kucinski et al., 2015). The reason for this effect in this study is currently unclear; however, rodent microdialysis studies have shown that ACh increases during periods of increased attention (Moore et al., 1992; Patel et al., 2012). The potential additional attentional load induced by the distractor may have increased ACh, which in turn improved aspects of complex motor control. This effect would depend on the availability of endogenous ACh and it would be interesting to repeat this in a disease model with a cholinergic deficit. We also only tested this relatively mild form of distraction and a different outcome may be seen with more attentionally demanding stimuli such as a predator scent.

## Acute cholinergic but not dopaminergic antagonism induces a deficit in complex motor performance

Scopolamine increased foot slips, traversal time and composite score, indicating that acute muscarinic antagonism impairs motor control in this task. These results are consistent with both clinical and preclinical studies. Reduced cholinergic transmission has also been reported in PD patients who have suffered a fall compared to healthy older subjects (Pelosin et al., 2016). Rats with bilateral cortical cholinergic lesions had an increased rate of falls in the MCMCT without affecting control measures such as basic limb co-ordination (Kucinski et al., 2013). While the same controls were not conducted in this study, previous work has shown that scopolamine at the same doses induces hyperlocomotion in measures of general activity (Bushnell, 1987). This suggests that slowing in traversal time induced by scopolamine is specific to complex motor control. Furthermore, we show a rod size by drug interaction, suggesting that scopolamine has specific effects related to attentional load, rather than a non-specific impairment of general activity. It should be acknowledged that there is the potential for systemic administration of scopolamine to induce non-specific side effects, and conflicting effects of cholinergic release and/or blockade at the nicotinic versus muscarinic receptors. However, as described above, our results align with previous work utilising a more specific approach (Kucinski et al., 2013), suggesting these potential confounding factors are not driving the results in this study. Cholinergic antagonism may disrupt cortico-striatal information transfer. In the context of attention, cholinergic transients are required to act on behaviourally relevant cues, such as a slip which would require a corrective movement. These are detected by synapses on striatal medium spiny neurons, which project to the substantia nigra and globus pallidus. It has been shown that cues are detected at a very low rate following cholinergic loss, therefore depriving the striatum of information (Sarter et al., 2014). It has been shown that muscarinic acetylcholine receptors are essential for transferring cortical cholinergic activity to the striatum, and therefore, receptor antagonism may reduce communication between the cortex and striatum (Nelson et al., 2005).

Complex motor impairments observed in the scopolamine study only occurred at either the 10- or 8-mm rod. Similar to the findings with the distractor, it may be that the wider rods require less attentional resource, while at more challenging dimensions, increased endogenous ACh interacts to reduce the impacts of scopolamine. This highlights the importance of designing a task with the optimum attentional load. Higher doses of scopolamine may be necessary to induce an impairment on the 6-mm rod to overcome the potential rise in acetylcholine.

Administration of alpha-flupentixol had no effect on complex motor performance in our task. This lack of effect is consistent with a previous study showing that partial striatal dopaminergic deafferentation had little to no effect on performance in the MCMCT (Kucinski et al., 2013; Kucinski and Sarter, 2015). It has been suggested that the motor deficits of striatal dopaminergic disruption in PD patients and the healthy elderly can be ‘masked’ by the compensatory cortical cholinergic system by allocating greater attentional resources to gait and posture. As such, inducing loss in both systems may unmask the impact of striatal dopaminergic disruption (Sarter et al., 2014). It was found that dual lesioning of the cortical cholinergic and striatal dopaminergic system increased falls, slowed traversal speed and impaired active rebalancing in the MCMCT compared to cholinergic lesions alone (Kucinski et al., 2013). In contrast, we show that while the combination of both scopolamine and alpha-flupentixol impaired complex motor control compared to vehicle, it did not potentiate the deficit observed with scopolamine alone. The scopolamine dose chosen may have created a ceiling effect whereby differences in scopolamine and scopolamine plus alpha-flupentixol could not be observed. Future work should consider using lower doses of scopolamine to investigate this further. It should be noted that the reduction in traversal time in the scopolamine dose–response study did not occur in the scopolamine plus alpha-flupentixol study. Traversal time may become less sensitive to cholinergic antagonism over repeat sessions and this should be considered when the order of experiments is being designed. However, it is clear that scopolamine impairs complex motor performance, and together with previous work highlights the cholinergic system as a therapeutic target for the complex motor deficits in PD. Promising clinical benefits have been found that the anticholinesterase drug Rivastigmine improved gait stability in patients with PD in phase 2 trials (Henderson et al., 2016).

Testing the effects of both scopolamine and alpha-flupentixol in the presence of a distractor would be an interesting next step. However, our results show that careful consideration of the correct attentional load, drug dose and distractor type is required to unpick this interaction. Therefore, more initial work needs to be done before we can meaningfully test an interaction between the cholinergic/dopaminergic antagonism and attention in this task.

While it is not possible to directly compare performance between the aged mice and those treated with scopolamine as they were separate experiments, aged mice seem to show a greater deficit in the task. This suggests that while acute pharmacological manipulation at the doses chosen can induce impairment, it is not to the same extent as a model with multi-system changes. Cholinergic disruption is generally more severe in PD patients compared to healthy older people (Pelosin et al., 2016; Sarter et al., 2014). As such, use of a genetic mouse model of PD which captures multi-system degeneration would be a valuable method to assess the full impact of cholinergic and dopaminergic disruption.

It is important to note that while this study utilised male mice for the ageing studies and female mice for the pharmacological studies, the use of both sexes in both parts of the work would benefit the overall translatability of our findings.

## Conclusion

Aged mice showed an increased number of foot slips, a slower traversal time, a greater number of falls and a higher composite score compared to younger mice, which may suggest a complex motor control deficit, consistent with the clinical literature. Rimadyl reduced number of foot slips, indicating that this aspect of motor control may be related to inflammation, and is dissociable from other measures. Scopolamine increased foot slips, traversal time and composite score, which may indicate a reduction in complex motor control induced by a reduction in cholinergic activity. These effects were not seen using alpha-flupentixol. A combined approach increased foot slips and composite score relative to vehicle but did not potentiate effects seen with scopolamine alone, which contrasts with previous work. The use of a mild attentional distractor did not yield the predicted effects and seemed to improve performance in aged mice. Together, these data provide preliminary evidence that this complex motor task design may represent a method relevant to motor impairments in PD and normal ageing. Testing of different distractors is needed to determine whether this approach could be used to test the interaction between complex motor control and attention. However, our findings are largely consistent with prior work showing the importance of the cholinergic system and attentional load in complex motor control and the need to design future therapies with this in mind.
